# Sleep behaviors modify the association between hemoglobin concentration and respiratory infection: a prospective cohort analysis

**DOI:** 10.3389/fphys.2025.1638819

**Published:** 2025-09-30

**Authors:** Yongkui Zhu, Qian Chen, Mengying Wang, Huan Qian, Qiying Song, Bofei Liu

**Affiliations:** ^1^ Department of Intensive Care Unit, The Affiliated Zhangjiagang Hospital of Soochow University, Suzhou, China; ^2^ Department of Nutrition and Food Hygiene, School of Public Health, Peking University, Beijing, China; ^3^ Key Laboratory of Epidemiology of Major Diseases (Peking University), Ministry of Education, Beijing, China; ^4^ Department of Anesthesia, Zhangjiagang Hospital of Traditional Medicine, Suzhou, China; ^5^ Department of Child Healthcare, Shenzhen Baoan Women’s and Children’s Hospital, Shenzhen, China

**Keywords:** sleep behaviors, hemoglobin concentration, respiratory infection, cohort, interaction

## Abstract

**Interpretation:**

Hb and respiratory infection showed a nonlinear U-shaped association; such a relation is modified by the chronotype sleep behavior.

**Objective:**

To examine the association between Hb and the incidence of hospitalized respiratory infection, and to explore potential modification effects of sleep behaviors.

**Methods:**

Included were 292,568 individuals without respiratory disease, cancer, or anemia diagnosis in the United Kingdom Biobank . Hb (g/dL) was measured at baseline. The interaction between Hb and sleep behaviors, including sleep duration, insomnia, chronotype, and daytime sleepiness with respiratory infection, was tested.

**Results:**

The cohort was followed up at a median 12.6 years, and 16,669 incident respiratory infections (9,334 in men, 7,335 in women) were identified. There was a nonlinear U-shaped association between Hb and respiratory infection in both men and women, where the risk increased markedly with Hb above 15.0 g/dL for men and 13.5 g/dL for women. In men, compared with the third quintile group, the hazard ratio (HR; 95% confidence interval [CI]) of respiratory infection in the Q1, Q2, Q4, and Q5 quintile groups was 1.28 (1.21–1.37), 1.07 (1.00–1.14), 1.06 (0.99–1.13), and 1.09 (1.02–1.17), respectively. In women, the HR (95% CI) was 1.20 (1.12–1.29), 1.09 (1.01–1.17), 1.01 (0.94–1.09), and 1.05 (0.98–1.13) in the Q1, Q2, Q4, and Q5 quintile groups of Hb, respectively, compared with the third quintile group. There was a significant interaction between Hb concentration and chronotype on the risk of respiratory infection (*P* for interaction = 0.005). The elevated risk of respiratory infection associated with Hb was more pronounced among participants with late chronotype.

**Conclusion:**

The study suggests that Hb and respiratory infection have a nonlinear U-shaped association and that such a relation is modified by chronotype.

## Introduction

Hemoglobin (Hb) is an iron-containing protein responsible for oxygen transport in red blood cells (Ciaccio et al., 2022). Lower Hb concentrations have been recognized as an indicator of anemia (Stevens et al., 2013). Symptoms including fatigue, weakness, and cognitive decline may originate from anemia, in which tissue oxygen delivery is limited (Schumann and Solomons, 2017). Globally, approximately 1.93 billion people had anemia (Kassebaum and Collaborators, 2016). Previous studies have shown that Hb concentration is associated with adverse health outcomes including infections, chronic heart failure, and mortality (Harrison et al., 2021; K et al., 2020; Gelaw et al., 2021). Of note, respiratory infections are one of the most common causes of mortality and disability-adjusted life-years worldwide, especially during the coronavirus disease 2019 (COVID-19) pandemic (Diseases and Injuries, 2020; Williamson et al., 2020). Lower respiratory infections have high morbidity and mortality rates and contribute to a substantial burden on individuals, families, and the society (GBD 2016 Lower Respiratory Infections Collaborators, 2018). Recent meta-analyses have evaluated the association between Hb and respiratory infection (Taneri et al., 2020; Kowsar et al., 2023), but conflicting results were reported. For example, a meta-analysis found that lower concentrations of Hb were associated with higher mortality in COVID-19 patients (Kowsar et al., 2023). However, Taneri et al. (2020) observed a non-significant difference in Hb concentrations between survivors and non-survivors among COVID-19 patients. An observational study also showed that Hb did not differ significantly between critically ill and moderate COVID-19 patients (Zeng et al., 2020). In addition, the sex-specific dose response association between Hb concentration and respiratory infection risk remains poorly explored in large-scale cohorts, despite sex differences in anemia prevalence and respiratory infection risk being documented (Levi et al., 2019; Zhang et al., 2022). Considering the physiological differences in Hb concentration thresholds, immune function, and respiratory disease susceptibility between men and women (Levi et al., 2019; Zhang et al., 2022), the current study specifically addresses this gap using a large, longitudinal cohort.

Previous studies have shown that the modification effects of various lifestyle factors might partly explain inconsistent associations between biomarkers and health outcomes (Wang et al., 2020; Wang et al., 2022). Notably, sleep behaviors are emerging lifestyle factors that are closely related to both Hb and respiratory infection (Jackowska et al., 2013; Chen et al., 2020; Shafiee et al., 2023; Chiner et al., 2016; Chen et al., 2021). Population-based investigations have shown that sleep duration and disturbance were related to Hb concentration (Jackowska et al., 2013; Chen et al., 2020). Furthermore, sleep disturbance was related to elevated risk of respiratory infection (Shafiee et al., 2023; Chiner et al., 2016; Chen et al., 2021). A recently published study showed that obstructive sleep apnea was associated with severe COVID-19 and longer hospitalization (Arish et al., 2023). Therefore, sleep behaviors might be potential modifiers in the association between Hb and the risk of respiratory infection. However, there has been limited investigation into the role of sleep behaviors in the relationship between Hb and respiratory infection.

Using data from the United Kingdom Biobank (UKB), we tested the sex-specific dose–response association between Hb and the risk of any hospitalized respiratory infection incidence and explored the potential modification effects of several sleep behaviors, including excessive daytime sleepiness, insomnia, sleep duration, and chronotype.

## Methods

### Study design and population

The detailed study design and participants of UKB have been described in Sudlow et al. (2015). In brief, more than 500,000 community-based volunteers aged 37–73 years across the United Kingdom were enrolled between 2006 and 2010. All participants completed touchscreen questionnaires and physical measurements during a baseline survey. They also provided blood samples for hematology and biomarker testing. The follow-up information for all participants was mainly obtained through record linkage to national electronic health data, including death and cancer registers, primary care, and hospital in-patient. All participants gave written consent, and ethical approval was obtained from the North-West Multi-Centre Research Ethics Committee (Ref 11/NW/0382).

For the current analysis, participants with any respiratory disease, cancer, or anemia diagnosis were excluded (n = 27 258). Those without Hb measurements (n = 51 634) or sleep variables (n = 74 303) at baseline were also excluded, leaving a total of 292 568 individuals in the analysis.

### Assessment of hemoglobin concentration

Blood samples were collected from participants for hematology analysis during baseline visit. Four Beckman Coulter LH750 instruments were used to analyze samples for hematology data. The detailed information on this data collection is provided at the UKB website (https://biobank.ndph.ox.ac.uk/showcase/refer.cgi?id=1453).

### Assessment of sleep behaviors

Sleep behaviors data were collected through a self-reported touchscreen questionnaire in the UKB. Details about questions of sleep behaviors were described by Fan et al. (2019). In the current study, only four sleep behaviors (excessive daytime sleepiness, sleep duration, chronotype, insomnia) were included since snoring was closely related to respiratory diseases. Each sleep behavior was classified as low- or high-risk and coded as “1” or “0”, respectively. Low-risk sleep behaviors were defined according to the following criteria: sleep 7–8 h per day; early chronotype (“morning” or “morning than evening”); never or rarely reported insomnia symptoms; no excessive daytime sleepiness (“never/rarely” or “sometimes”). High risk sleep behaviors referred to sleep less than 7 h/day or more than 8 h/day, late chronotype (“evening than morning” or “evening”), sometimes or usually reported insomnia symptoms, and excessive daytime sleepiness (“often” or “all of the time”).

### Assessment of outcomes

Health outcomes in the UKB were defined based on multiple resources of records including death register, cancer register, hospital admissions data, and self-report information. Hospital admission data were obtained through Scottish Morbidity Record data in Scotland, Hospital Episode Statistics in England, and the Patient Episode Database in Wales. Any incident hospitalized respiratory infection was ascertained with the International Statistical Classification of Diseases and Related Health Problems, Tenth Revision (ICD-10) codes of J00, J01, J02, J03, J04, J05, J06, J09, J10, J11, J12, J13, J14, J15, J16, J17, J18, J20, J21, and J22. Specifically, J00, J01, J02, J03, J04, J05, J06, J09, J10, and J11 were identified as upper respiratory infections, while J12, J13, J14, J15, J16, J17, J18, J20, J21, and J22 were lower respiratory infections.

### Statistical analysis

Male and female participants were separately classified into five groups according to quintiles of Hb concentrations. For the baseline characteristics of participants, continuous and categorical variables were described as means or percentages, respectively, according to the quintile groups. The period between the baseline date and the first occurrence of any respiratory infection diagnosis, death, or the census date (30 September 2021) was estimated as the follow-up time. The Cox proportional hazards model was adopted to calculate the sex-specific hazard ratio (HR) and 95% confidence interval (CI) of respiratory infection, with the third quintile as the reference group. In the first model, adjustments were made for demographics including age, race (white European, mixed, South Asian, black, others), Townsend deprivation index, and UKB assessment center. Model 2 was further adjusted for lifestyle factors such as alcohol consumption (never, former, current), smoking status (never, former, current), healthy diet score (0–5), iron supplement intake (yes/no), and body mass index (BMI, kg/m^2^). In model 3, we further adjusted for hypertension (yes/no), diabetes (yes/no), and cardiovascular disease (yes/no) at baseline. We tested the proportional hazards assumption using the Schoenfeld residuals method, and the models were satisfied for Hb. The healthy diet score was calculated by using five dietary factors: fruits, vegetables, unprocessed red meat, processed meat, and fish. Each was classified as a low risk factor and scored one point according to the favorable corresponding median values: vegetables at least four tablespoons/day; fruits at least three pieces/day; fish at least twice/week; unprocessed red meat no more than twice/week; processed meat no more than twice/week. The total healthy diet score ranged from 0 to 5 (Pazoki et al., 2018). Restricted cubic splines were constructed to assess the association of Hb with respiratory infection based on model 3. Notably, quintile-based categorization of Hb concentration was adopted for maintaining relatively sufficient statistical power and capturing the nonlinear association between Hb and respiratory infection risk. For missing continuous variables, mean values were imputed. For missing categorical variables, a missing indicator approach was used. The missing rate of race, alcohol consumption, and smoking status was 0.3%, 0.9%, and 0.3%, respectively. The likelihood ratio test comparing models with and without a cross-product term of Hb and each sleep factor was conducted to test the interaction between Hb and sleep behaviors. All sleep behaviors were included in models simultaneously.

Sensitivity analyses with restricting incident respiratory infection cases to more than 2 years from the baseline survey time or excluding participants with extreme hemoglobin values (physiologically plausible range: 12–16 g/dL for men and 11–15 g/dL for women) were conducted to confirm the robustness of the results.

SAS software (version 9.4; SAS Institute Inc., Cary, NC, United States) and R (version 4.5.1) were used to conduct the analyses. A two-sided *P*-value less than 0.05 was considered statistical significant.

## Results

### Baseline characteristics

Baseline characteristics of the study populations according to quintiles of Hb concentrations are shown in Table 1. Among all participants included in the current analysis, 46.3% (135 351 individuals) were male. The mean (standard deviation [SD]) Hb concentrations were 15.0 (1.0) and 13.5 (0.9) g/dL for men and women, respectively. Participants with higher Hb concentrations were older, had higher BMI levels, tended to be current smokers, but were less likely to have a healthy diet. Furthermore, participants with higher Hb concentrations also tended to have hypertension but had a lower prevalence of iron supplement intake than those with lower Hb concentrations.

**TABLE 1:** Baseline characteristics of participants according to quintiles of serum hemoglobin concentration.

Characteristics	Serum hemoglobin concentration quintiles, g/dL				
	Q1	Q2	Q3	Q4	Q5
N	58 294	55 447	61 149	59 065	58 613
Men	26 879	25 704	28 419	27 201	27 148
Women	31 415	29 743	32 730	31 864	31 465
Hemoglobin concentrations (mean, g/dL)					
Men	13.6	14.5	15.0	15.5	16.4
Women	12.2	13.0	13.5	14.0	14.8
Age (years)	55.7	56.0	56.1	56.3	56.8
Race, white (%)	91.4	94.4	95.2	95.8	96.1
Townsend deprivation index	−1.2	−1.4	−1.5	−1.5	−1.5
Body mass index (kg/m^2^)	26.6	26.9	27.2	27.5	28.1
Current drinkers (%)	90.9	93.1	93.5	93.4	93.1
Current smokers (%)	8.2	8.8	9.8	10.8	14.7
Healthy diet score					
0–1	10.8	11.1	11.8	12.2	13.6
2–3	48.5	48.3	48.2	49.0	48.9
4–5	40.7	40.5	40.0	38.8	37.5
Hypertension (%)	46.4	48.6	52.1	55.8	63.7
Diabetes (%)	6.9	4.4	4.1	3.8	4.1
Cardiovascular disease (%)	7.2	6.0	5.5	5.4	5.5
Sleep behaviors (low risk, %)					
Sleep duration	68.6	69.7	69.6	69.4	68.3
Chronotype	63.1	63.2	62.9	63.1	62.8
Daytime sleepiness	97.1	97.6	97.7	97.8	97.7
Insomnia	74.0	73.9	74.1	73.9	72.9
Iron supplement intake (%)	4.0	2.8	2.5	2.3	2.0

### Sex-specific associations between Hb concentrations and respiratory infections

**FIGURE 1:**
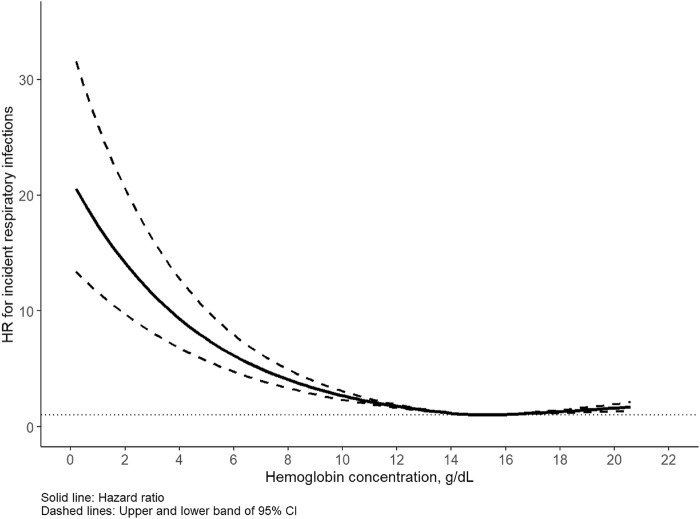
Association between hemoglobin and respiratory infection in males. Hazard ratio (HR) adjusted for age, race, United Kingdom Biobank assessment center, Townsend deprivation index, alcohol consumption, smoking status, healthy diet score, body mass index, iron supplement intake, hypertension, diabetes, cardiovascular disease.

**FIGURE 2:**
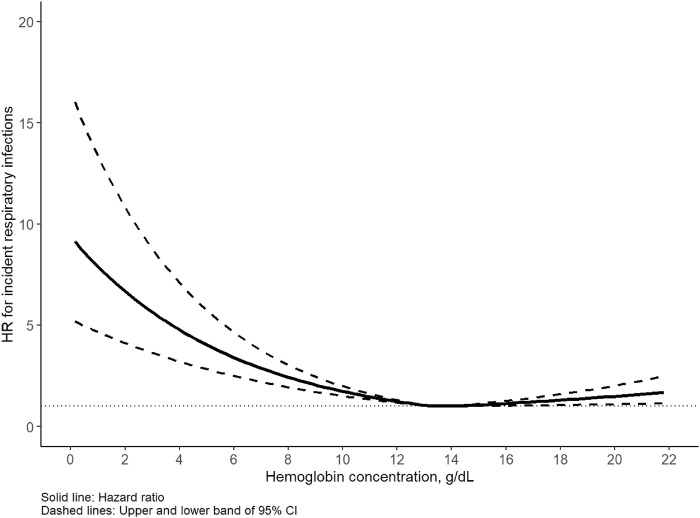
Association between hemoglobin and respiratory infection in females. Hazard ratio (HR) adjusted for age, race, United Kingdom Biobank assessment center, Townsend deprivation index, alcohol consumption, smoking status, healthy diet score, body mass index, iron supplement intake, hypertension, diabetes, cardiovascular disease.

The median follow-up time of the cohort was 12.6 years, during which 16,669 incident respiratory infections (9,334 in males, 7,335 in females) were identified. The incidence rate of any respiratory infection was 5.7/1,000 person-years for men and 3.8/1,000 person-years for women. Of note, the corresponding cases of upper and lower respiratory infections were 2067 (12.4%) and 14,602 (87.6%), respectively. A U-shaped association was detected between Hb concentration and respiratory infection for all participants as well as for men and women separately (Figures 1, 2). Statistically significant nonlinear associations between Hb concentration and respiratory infection risk were observed in both men (*P* for nonlinear <0.01) and women (*P* for nonlinear <0.01). The C-index for the restricted cubic spline was 0.70 in males and 0.68 in females, respectively. The risk increased markedly with Hb concentration above 15.0 g/dL in males and 13.5 g/dL in females.

In all multivariable-adjusted models, compared with the third quintile group, other quintile groups were associated with higher risks of respiratory infection incidents. In model 3 with adjustment for sex, age, race, Townsend deprivation index, UKB assessment center, alcohol intake, smoking, healthy diet score, BMI, iron supplement intake, hypertension, diabetes, and cardiovascular disease, the HR (95% CI) was 1.25 (1.20–1.32), 1.08 (1.02–1.13), 1.04 (0.99–1.09), and 1.07 (1.02–1.12), respectively, in the first, second, fourth, and fifth quintile groups compared with the third quintile group of Hb concentration. In men, the HR (95% CI) was 1.28 (1.21–1.37), 1.07 (1.00–1.14), 1.06 (0.99–1.13), and 1.09 (1.02–1.17), respectively, in the first, second, fourth, and fifth quintile groups compared with the third quintile group of Hb concentration. In females, the corresponding HR (95% CI) was 1.20 (1.12–1.29), 1.09 (1.01–1.17), 1.01 (0.94–1.09), and 1.05 (0.98–1.13) (Table 2). However, no statistically significant interaction between Hb concentration and sex on respiratory infection was observed in the current analysis (*P* for interaction >0.05).

**TABLE 2:** Sex-specific adjusted HRs and 95% CI for hemoglobin concentrations with respiratory infections.

Study populations		Serum hemoglobin concentrations (quintiles) concentrations				
		Q1	Q2	Q3	Q4	Q5
All participants	Cases/N	3815/58 294	3023/55 447	3128/61 149	3189/59 065	3514/58 613
	Model 1^a^	1.27 (1.21–1.33)	1.07 (1.01–1.12)	1.00	1.06 (1.00–1.11)	1.15 (1.09–1.21)
	*P*	<0.01	0.01	—	0.03	<0.01
	Model 2^b^	1.30 (1.24–1.36)	1.08 (1.03–1.14)	1.00	1.03 (0.98–1.08)	1.07 (1.02–1.12)
	*P*	<0.01	<0.01	—	0.21	0.01
	Model 3^c^	1.25 (1.20–1.32)	1.08 (1.02–1.13)	1.00	1.04 (0.99–1.09)	1.07 (1.02–1.12)
	*P*	<0.01	<0.01	—	0.16	<0.01
Men	Cases/N	2330/26 879	1693/25 704	1721/28 419	1740/27 201	1850/27 148
	Model 1^a^	1.30 (1.22–1.38)	1.06 (0.99–1.13)	1.00	1.07 (100–1.15)	1.15 (1.08–1.23)
	*P*	<0.01	0.11	—	0.04	<0.01
	Model 2^b^	1.33 (1.25–1.42)	1.07 (1.00–1.15)	1.00	1.05 (0.98–1.12)	1.08 (1.01–1.16)
	*P*	<0.01	0.04	—	0.14	0.02
	Model 3^c^	1.28 (1.21–1.37)	1.07 (1.00–1.14)	1.00	1.06 (0.99–1.13)	1.09 (1.02–1.17)
	*P*	<0.01	0.06	—	0.10	0.01
Women	Cases/N	1485/31 415	1330/29 743	1407/32 730	1449/31 864	1664/31 465
	Model 1^a^	1.20 (1.11–1.29)	1.07 (1.00–1.16)	1.00	1.04 (0.97–1.12)	1.16 (1.08–1.24)
	*P*	<0.01	0.06	—	0.30	<0.01
	Model 2^b^	1.23 (1.14–1.32)	1.10 (1.02–1.18)	1.00	1.01 (0.94–1.09)	1.05 (0.98–1.13)
	*P*	<0.01	0.02	—	0.73	0.18
	Model 3^c^	1.20 (1.12–1.29)	1.09 (1.01–1.17)	1.00	1.01 (0.94–1.09)	1.05 (0.98–1.13)
	*P*	<0.01	0.03	—	0.74	0.19

^a^ Adjusted for age, race, United Kingdom, Biobank assessment center, Townsend deprivation index.

^b^ Adjusted for age, race, United Kingdom, Biobank assessment center, Townsend deprivation index, alcohol consumption, smoking status, healthy diet score, body mass index, iron supplement intake.

^c^ Adjusted for age, race, United Kingdom, Biobank assessment center, Townsend deprivation index, alcohol consumption, smoking status, healthy diet score, body mass index, iron supplement intake, hypertension, diabetes, cardiovascular disease.

HR, hazard ratio; CI, confidence interval.

### Associations between sleep behaviors and respiratory infections

Associations between sleep behaviors and respiratory infections are shown in Table 3. We found that all healthy sleep behaviors were associated with lower risks of respiratory infections. In detail, the HR (95% CI) of respiratory infection was 0.89 (0.86–0.92) in the low-risk group of sleep duration compared with the high-risk group. In addition, the low-risk groups for chronotype, insomnia, and daytime sleepiness were associated with 4%, 9%, and 12% lower risk of respiratory infection, respectively.

**TABLE 3:** Adjusted^a^ HRs and 95% CI for sleep behaviors with respiratory infections.

Variables		Cases/N	HR (95% CI)	*P*
Sleep duration	High risk	6098/90 342	1.00	—
	Low risk	10 571/202 226	0.89 (0.86–0.92)	<0.001
Chronotype	High risk	6273/108 124	1.00	—
	Low risk	10 396/184 444	0.96 (0.93–0.99)	0.007
Insomnia	High risk	5115/76 837	1.00	—
	Low risk	11 554/215 731	0.91 (0.88–0.95)	<0.001
Daytime sleepiness	High risk	612/7093	1.00	—
	Low risk	16 057/285 475	0.88 (0.81–0.96)	0.002

^a^ Adjusted for age, sex, race, United Kingdom, Biobank assessment center, Townsend deprivation index, alcohol consumption, smoking status, healthy diet score, body mass index, hypertension, diabetes, cardiovascular disease.

HR, hazard ratio; CI, confidence interval.

### Interactions between Hb and sleep behaviors with respiratory infections

**FIGURE 3:**
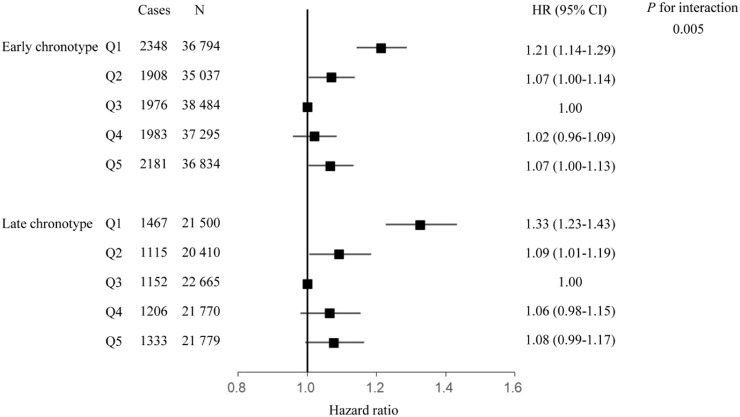
Association between hemoglobin and respiratory infection, stratified by chronotype. Hazard ratio adjusted for age, race, United Kingdom Biobank assessment center, Townsend deprivation index, alcohol consumption, smoking status, healthy diet score, body mass index, iron supplement intake, hypertension, diabetes, cardiovascular disease, sleep duration, insomnia, daytime sleepiness.

Interaction and subgroup analyses according to sleep factors were conducted to evaluate whether sleep behaviors modified the relationship between Hb and the risk of respiratory infection. There was a significant interaction between Hb concentration and chronotype for the risk of incident respiratory infection (*P* for interaction = 0.005); the HR (95% CI) was 1.32 (1.22, 1.42), 1.09 (1.00, 1.18), 1.07 (0.99, 1.16), and 1.08 (1.00, 1.17), respectively, in the first, second, fourth, and fifth quintile groups compared with the third quintile group of Hb concentration when further adjusted for the cross-product term of Hb and chronotype in the model. Subgroup analysis showed that the elevated HR of respiratory infection associated with Hb was more evident among participants in the high-risk chronotype group than in the low-risk group. The HR (95% CI) of respiratory infection incidents in first, second, fourth, and fifth quintile groups of Hb concentration was 1.33 (1.23–1.43), 1.09 (1.01–1.19), 1.06 (0.98–1.15), and 1.08 (0.99–1.17), respectively, among participants with late chronotype, and 1.21 (1.14–1.29), 1.07 (1.00–1.14), 1.02 (0.96–1.09), and 1.07 (1.00–1.13), respectively, among participants with early chronotype (Figure 3). The interactions between Hb concentration and other sleep behaviors did not show statistical significance.

### Sensitivity analysis

Sensitivity analysis showed that the results were generally stable when excluding participants with a follow-up time ≤2 years or those with extreme hemoglobin values. The results are shown in Supplementary Tables S1, S2.

## Discussion

In UKB, we observed a U-shaped association between Hb concentration and any incidence of hospitalized respiratory infection in which the risk of respiratory infection increased markedly with Hb above 15.0 g/dL for men and 13.5 g/dL for women. Significant associations of four sleep behaviors—excessive daytime sleepiness, sleep duration, chronotype, and insomnia—with respiratory infections were also observed. We also found that the association between Hb and respiratory infection was significantly modified by chronotype, and the higher risk of respiratory infection associated with either lower or higher Hb concentration appeared to be stronger among participants with late chronotype.

The present study showed that the association between Hb and respiratory infection was nonlinear and U-shaped, with either low or high Hb concentration being associated with a higher risk of respiratory infection. Patino-Aldana et al. (2022) found a nonlinear, U-shaped association between Hb concentration and the risk of COVID-19 mortality similar to our results. Furthermore, a U-shaped association was also observed between serum iron and severe respiratory failure in COVID-19 patients (Tojo et al., 2021). The U-shaped association between Hb concentration and the risk of respiratory infection is biologically explainable. Hb concentration reflects the oxygen-carrying capacity of the blood (Hayden et al., 2012), and hypoxia may exert an adverse effect on immune responses through the NF-κB-driven inflammation pathway (Taylor et al., 2014) and programmed death-ligand 1 (PD-L1)/PD-1 crosstalk (Cubillos-Zapata et al., 2017). At the same time, higher Hb concentration might cause excess iron. Animal studies have shown that iron overload might cause lung injury due to apoptosis of the lung tissue, which consequently increases the risk of respiratory infection (Zhang et al., 2020; Liu et al., 2022). Additionally, the release of free iron from Hb might enhance oxidant damage in isolated rat lungs caused by t-buOOH (Seibert et al., 1991). The results illustrate the importance of achieving a delicate balance in iron intake in which neither too-low nor too-high Hb concentration has a negative health effect on respiratory tract. The proportion of lower respiratory infections is particularly higher than upper respiratory infections in the current analysis. Lower respiratory infections including pneumonia and bronchitis typically present with more severe symptoms, which may increase the likelihood of medical consultation and diagnosis. In contrast, milder upper respiratory infections may often remain self-managed and undiagnosed. This pattern is consistent with the case definition based on ICD codes in the UKB, which primarily captures clinically attended cases. Therefore, our findings may predominantly reflect the association between Hb and severe respiratory infections requiring medical attention rather than total community infections. The interpretation of our results should be addressed in future studies with more upper respiratory infection cases. Notably, no statistically significant interaction between sex and Hb concentration on respiratory infection was observed in the current study. However, biological differences in Hb concentration by sex is well-documented in clinical guidelines. Additionally, the current analysis also showed significantly different hemoglobin concentrations between sexes, with men having higher concentrations than women. Therefore, the results of the association between Hb concentration and respiratory infection were still presented by sex.

We also assessed the association between sleep behaviors and respiratory infection in the current analysis. The results were consistent with previous studies, which showed that unhealthy sleep behaviors might be related to a higher risk of respiratory infection (Cohen et al., 2009; Prather et al., 2015; Quan et al., 2023; Nieters et al., 2019). Like Hb, sleep also plays a vital role in maintaining optimal immune function (Besedovsky et al., 2012) which is strongly associated with respiratory infection (Garbarino et al., 2021). Several studies have demonstrated that sleep duration and sleep disturbances are related to the continuous production of inflammation markers, including pro-inflammatory cytokines, which might result in increased risks of inflammatory diseases (Garbarino et al., 2021; Irwin et al., 2016). Furthermore, insomnia is related to increased stress and the activation of the sympathetic nervous system, as well as elevated cortisol (Bonnet and Arand, 2010), which can inhibit the transcription of antiviral interferon genes through increased release of norepinephrine, thus inducing a state of chronic inflammation (Irwin and Opp, 2017; Furman et al., 2019). As emerging lifestyle risk factors, sleep behaviors—including no frequent excessive daytime sleepiness, sleep 7–8 h per day, no or rare insomnia, and early chronotype—have been reported to reduce the risks of cardiovascular diseases and type 2 diabetes in recent studies (Fan et al., 2019; Zhuang et al., 2023). The current study contributes to a more comprehensive picture of sleep behaviors and health outcomes.

Interestingly, in the interaction analysis, we observed that chronotype significantly modified the association between Hb concentration and respiratory infection risk. The finding of the interaction between chronotype and Hb seemed biologically plausible. It has been shown that Hb production might be disrupted by inflammation such as IL-6 (Mehta et al., 2020). Notably, inflammatory responses are gated by the circadian clock (Gibbs et al., 2012). An evening chronotype is associated with higher levels of circulating inflammatory markers (Kim et al., 2018). Therefore, the inflammatory pathway could partly explain the interaction between chronotype and Hb concentration for respiratory infection. The interaction analysis findings suggest that sleep behaviors are potential modifiers in the Hb-respiratory infection association and that the heterogeneity in associations between Hb and respiratory infection across different studies might be partly caused by sleep behaviors.

Smoking status should be an important consideration in interpreting results of the current study. Previous studies have shown that smoking is associated with both Hb concentration and susceptibility to respiratory infection (Pedersen et al., 2019; Jiang et al., 2020). Additionally, smoking may also contribute to a higher risk of sleep disturbance (Amiri and Behnezhad, 2020). In the current analysis, despite its adjustment for self-reported smoking status in the multivariable models, information bias and residual confounding may persist. Furthermore, smoking may act as an effect modifier in the hemoglobin–respiratory infection association, although no significant interaction was detected in the current analysis. Further studies are needed to clarify these complex pathways.

### Strengths and limitations

This study used UKB data to assess the sex-specific association between Hb and respiratory infection, and particularly to explore the interaction between Hb concentration and sleep behaviors with incident respiratory infection risk. The cohort study has a large sample size, longitudinal design, and valid measurements of Hb concentration. Additionally, a number of confounders including lifestyles, physical measurements, and medical records were adjusted to reduce potential bias. However, there were still limitations to be addressed. First, causality relationships could not be inferred since this was an observational study. Therefore, intervention studies and mechanistic experiments are needed to confirm and explain the findings. Second, although several important confounding variables were taken into account, some unmeasurable confounders might affect the association. Third, self-reported sleep data were adopted in the study, so misclassification of exposures might exist. Fourthly, only a single measurement of Hb concentration is available at baseline. Repeated measurements of Hb concentration are needed to confirm the association. Fifthly, ICD-10 codes were used to identify respiratory infections in the UKB, which may capture more severe infections recorded in clinical settings. Thus, the respiratory incidence rates should not be interpreted as estimates of total community infection incidence, and the results should be interpreted with caution. Additionally, in order to increase statistical efficiency, especially in stratified analyses, different subtypes of respiratory infection were not considered. Finally, more caution is needed when generalizing and interpreting the results to other populations since the participants were all enrolled in the United Kingdom.

## Conclusion

The study indicates that either lower or higher Hb concentration is associated with an elevated risk of hospitalized respiratory infection, indicating a U-shaped relation in both males and females. In addition, individual sleep behaviors including excessive daytime sleepiness, sleep duration, chronotype, and insomnia are related to the risk of hospitalized respiratory infection. More importantly, the association between Hb concentration and hospitalized respiratory infection is modified by chronotype. It is important to consider sleep behaviors when investigating the association between Hb and hospitalized respiratory infection.

## Data Availability

The raw data supporting the conclusions of this article will be made available by the authors without undue reservation.
