# Supplementary material for: Sleep behaviors modify the association between hemoglobin concentration and respiratory infection: a prospective cohort analysis
Source: Front Physiol. 2025 Sep 30;16:1638819. doi: 10.3389/fphys.2025.1638819 (PMC12518324; doi:10.3389/fphys.2025.1638819)
Supplement: Supplementary file 1 [file Table1.docx]

Supplementary Table 1 Sex-specific adjusted HRs and 95% CI for hemoglobin concentrations with respiratory infections after excluding participants with a follow-up time ≤ 2 years

|  |  | Serum hemoglobin concentrations (quintiles) concentrations | | | | |
| --- | --- | --- | --- | --- | --- | --- |
|  |  | Q1 | Q2 | Q3 | Q4 | Q5 |
| All participants | HR (95% CI) | 1.24 (1.18-1.30) | 1.07 (1.02-1.13) | 1.00 | 1.03 (0.98-1.08) | 1.07 (1.02-1.13) |
|  | *P* | <0.01 | <0.01 | - | 0.28 | <0.01 |
| Men | HR (95% CI) | 1.26 (1.18-1.34) | 1.07 (0.99-1.14) | 1.00 | 1.05 (0.98-1.12) | 1.09 (1.02-1.17) |
|  | *P* | <0.01 | 0.07 | - | 0.21 | 0.01 |
| Women | HR (95% CI) | 1.20 (1.11-1.29) | 1.08 (1.00-1.17) | 1.00 | 1.01 (0.94-1.09) | 1.06 (0.98-1.14) |
|  | *P* | <0.01 | 0.05 | - | 0.76 | 0.14 |

Adjusted for age, sex, race, UK Biobank assessment centre, Townsend Deprivation index, alcohol consumption, smoking status, healthy diet score, body mass index, iron supplement intake, hypertension, diabetes, and cardiovascular disease.

HR, hazard ratio; CI, confidence interval

Supplementary Table 2 Sex-specific adjusted HRs and 95% CI for hemoglobin concentrations with respiratory infections after excluding participants with extreme hemoglobin values

|  |  | Serum hemoglobin concentrations (quintiles) concentrations | | | | |
| --- | --- | --- | --- | --- | --- | --- |
|  |  | Q1 | Q2 | Q3 | Q4 | Q5 |
| All participants | HR (95% CI) | 1.22 (1.16-1.28) | 1.08 (1.02-1.13) | 1.00 | 1.04 (0.99-1.09) | 1.03 (0.97-1.09) |
|  | *P* | <0.01 | <0.01 | - | 0.16 | 0.40 |
| Men | HR (95% CI) | 1.25 (1.17-1.33) | 1.07 (1.00-1.14) | 1.00 | 1.06 (0.99-1.13) | 1.02 (0.92-1.13) |
|  | *P* | <0.01 | 0.06 | - | 0.10 | 0.77 |
| Women | HR (95% CI) | 1.17 (1.19-1.26) | 1.09 (1.01-1.17) | 1.00 | 1.01 (0.94-1.09) | 1.03 (0.96-1.12) |
|  | *P* | <0.01 | 0.03 | - | 0.73 | 0.41 |

Adjusted for age, sex, race, UK Biobank assessment centre, Townsend Deprivation index, alcohol consumption, smoking status, healthy diet score, body mass index, iron supplement intake, hypertension, diabetes, and cardiovascular disease.

HR, hazard ratio; CI, confidence interval
